# Integrated Analysis of circRNA-miRNA-mRNA-Mediated Network and Its Potential Function in Atrial Fibrillation

**DOI:** 10.3389/fcvm.2022.883205

**Published:** 2022-06-30

**Authors:** Feiyu Wei, Xi Zhang, Xiaohui Kuang, Xiaolong Gao, Jing Wang, Jie Fan

**Affiliations:** ^1^Faculty of Life Science and Technology, Kunming University of Science and Technology, Kunming, China; ^2^Department of Cardiology, The First People’s Hospital of Yunnan Province, The Affiliated Hospital of Kunming University of Science and Technology, Kunming, China

**Keywords:** atrial fibrillation, competitive endogenous RNA, circRNA, atrial fibrosis, prognosis

## Abstract

**Background:**

Atrial fibrillation (AF) is one of the most prevalent arrhythmias, characterized by a high risk of heart failure and embolic stroke. Competing endogenous RNA network has been reported to play an important role in cardiovascular diseases. The main objective of the present study was to construct a circRNA–miRNA–mRNA-mediated network and explore the potential function in AF.

**Methods:**

The microarray data of circRNA, miRNA, and mRNA in AF were downloaded from the Gene Expression Omnibus database. The RobustRankAggreg method was used to screen the different expression circRNAs(DECs). Then the circRNA–miRNA–mRNA-mediated network was constructed by using the CircInteractome database and the miRWalk online tool. A quantitative real-time polymerase chain reaction was used to detect the circRNA expression level in plasma. The left atrial fibrosis was evaluated with the left atrial low voltage area (LVA) by using left atrial voltage matrix mapping.

**Results:**

Three DECs (hsa_circRNA_102461, hsa_circRNA_103693, and hsa_circRNA_059880) and 4 miRNAs were screened. Then a circRNA–miRNA–mRNA-mediated network was constructed, which included 2 circRNAs, 4 miRNAs, and 83 genes. Furthermore, the plasma’s hsa_circ_0070391 expression level was confirmed to be upregulated and positively correlated with left atrial fibrosis in AF (*r* = 0.88, *P* < 0.001), whereas hsa_circ_0003935 was downregulated. Moreover, the ROC curve analysis revealed hsa_circ_0070391 and hsa_circ_0003935 could differentiate AF from the healthy controls with an AUC of 0.95 (95% sensitivity and 90% specificity) and 0.86 (70% sensitivity and 75% specificity), respectively. Finally, the free of atrial tachyarrhythmia rate was dramatically lower in the hsa_circ_0070391 high expression group than in the low expression group post catheter ablation (70.0 vs. 90.0%, *p* = 0.04).

**Conclusion:**

This study provides a novel insight to further understand the AF pathogenesis from the perspective of the circRNA–miRNA–mRNA network, suggesting that plasma circRNAs could serve as a novel atrial fibrosis and prognosis biomarker for AF.

## Introduction

Atrial fibrillation (AF) is a prevalent, sustained atrial arrhythmia mainly characterized by a high risk of embolic stroke (1). Radiofrequency catheter ablation was the first-line treatment with favorable outcomes for paroxysmal AF and persistent AF without major risks of recurrence (2). Atrial structural remodeling, which has been characterized as atrial fibrosis, is the primary pathological mechanism for AF development (3). Furthermore, the extent of pre-existing atrial fibrosis is obviously associated with the catheter ablation success rate (4). The extent of left atrial fibrosis can be evaluated by mapping low-voltage areas (LVAs) in the atrium using a multipolar mapping catheter or cardiac magnetic resonance imaging (5). However, non-invasive biomarkers of atrial fibrosis that can be used in preclinical settings would have great clinical benefits.

Circular ribonucleic acids (circRNAs) are a type of closed circular single-stranded RNA molecules connected by covalent bonds (6). Studies have demonstrated that circRNAs can serve as biomarkers for disease diagnosis and prognosis owing to plasma stability compared with linear transcripts (7). Previous studies have shown that circRNAs were associated with cardiac fibrosis and arrhythmia (7–9). In 2011, the concept of competitive endogenous RNA (ceRNA) was first proposed and subsequently supported by several studies, which described the competitive activity of some RNAs (as ceRNAs) by binding with target miRNAs, thereby altering the expression of target genes (10). The study has shown that circRNA mainly acts as ceRNA that regulates the expression of the miRNA target gene by competitively binding with miRNA (11).

Therefore, this study aimed to screen the circRNA–miRNA–mRNA network based on the publicly available gene microarray data and explore its potential function in AF. A circRNA-miRNA-mRNA network was developed, which included 2 circRNAs (hsa_circ_0003935 and hsa_circ_0070391), 4 miRNAs (hsa-miR-223, hsa-miR-630, hsa-miR-21, and hsa-miR-1305), and 83 mRNAs. Furthermore, the expression of hsa_circ_0070391 was confirmed to be upregulated and positively correlated with left atrial fibrosis. Finally, the free of atrial tachyarrhythmia rate was dramatically lower in the hsa_circ_0070391 high expression group than in the low expression group post catheter ablation (70.0 vs. 90.0%, *p* = 0.04). These results suggest that the circRNA–miRNA–mRNA network could serve as a potential non-invasive atrial fibrosis and prognosis biomarker for AF.

## Materials and Methods

### Data Collection

In this study, four microarray expression profiles (GSE129409, GSE97455, GSE28954, and GSE115574) were obtained from the Gene Expression Omnibus (GEO),^1^ details of which GEO datasets are shown in Supplementary Table 1. Both the GSE129409 (three AF samples and three normal samples) and GSE97455 (15 AF samples and 15 normal samples) datasets were circRNA expression profiles based on GPL21825 074301 Arraystar Human CircRNA microarray V2. The GSE28954 dataset contained miRNA expression profiles of 10 AF samples and 18 normal samples based on GPL10850 Agilent-021827 Human miRNA Microarray (V3) (miRBase release 12.0 miRNA ID version). A total of 28 AF samples and 31 normal samples based on GPL570 (HG-U133_Plus_2) Affymetrix Human Genome U133 Plus 2.0 Array were included in the GSE115574 dataset that consisted of mRNA expression profiles.

### Differential Expression Analysis

After the normalization and log2 transformation of the microarray data mentioned above, limma, an R software package, was used to identify DECs, DEMs, and DEGs between the AF and normal samples. The selection criterion for DECs was a *p* of < 0.05 and a | log_2_(fold change)| > 1, whereas the selection criterion for DEMs and DEGs was a *p* of < 0.01 and a | log2(fold change)| of > 1. The Robust rank aggregation package in R was used to integrate and rank all harvested DECs with the criterion of *p* < 0.05. The RRA method was based on the theory that genes in each experiment were randomly ordered (12). For the genes ranking higher in the experiment, the possibility of differential expression is inversely proportional to the value of *p*.

### Prediction of Different Expression Circular Ribonucleic Acids Target miRNAs

Target miRNAs of DECs were predicted using the Web tool circular RNA Interactome (CircInteractome)^2^ (Supplementary Tables 2–4). Predicted miRNAs from the CircInteractome dataset and DEMs were intersected through a Venn diagram to obtain miRNAs.

### Prediction of miRNA Target mRNAs

The miRWalk^3^ database was used to predict mRNAs of the overlapped miRNA (Supplementary Table 5). Overlapped genes between the miRNA target genes and DEGs were considered as final genes for further investigation.

### Establishment of the Circular Ribonucleic Acids–miRNA–mRNA Network

The ceRNA network was established by combining circRNA-miRNA and miRNA-mRNA interactions, and the regulatory network was visualized using the Cytoscape software.^4^

### Functional Enrichment Analysis

Gene Ontology (GO) annotation and Kyoto Encyclopedia of Genes and Genomes (KEGG) pathway analyses were performed on selected genes with the R package clusterProfler to explore biological functions and pathways. *p* < 0.05 was accepted as the cut-off criteria.

### Potential Treatment Drugs for Atrial Fibrillation

The selected target genes were imported into Connectivity Map (Cmap),^5^ to acquire small molecule drug prediction results with the criteria of *p* < 0.05,| enrichment| < 0.8. Moreover, the enrichment value was between −1 and 1, indicating the correlation between the input genes and small molecule drugs. Positive and negative numbers meant small molecule drugs induced or inhibited gene expression in the Cmap database, respectively. Then, the two-dimensional structures of the small molecule drugs were retrieved through the PubChem database.^6^

### Study Populations

This study included 80 patients with AF (40 persistent and 40 paroxysmal AF) who were consecutively hospitalized in our department from March 2020 to September 2020, and 40 individuals without AF evidence or history, who were hospitalized for palpitations and diagnosed as paroxysmal supraventricular tachycardia, were consecutive enrolled as controls during the same period. The diagnostic criteria for persistent AF and paroxysmal AF were according to the 2020 ESC guideline (2). The exclusion criteria were as follows: ages < 18 or > 80 years, severe underlying structural heart disease, contraindication for anticoagulation, serious liver or renal dysfunction, and LA or LAA thrombosis. This study was approved by the Ethics Committees of the First People’s Hospital of Yunnan Province. All participants signed a written informed consent document. All procedures were performed following the Declaration of Helsinki and relevant policies in China.

### Plasma Collection

Peripheral blood was collected using anti-ethylenediaminetetraacetic acid (EDTA) tubes and further extracted within 1 h. The blood sample was centrifuged at 2,000 *g* for 10 min, and the upper liquid was obtained. The solution was stored at 80°C for further experiments. Furthermore, a left atrial blood sample was collected after atrial septal puncture before heparin was used during the radiofrequency catheter ablation.

### RNA Extraction and Quantitative Reverse Transcription-Polymerase Chain Reaction

To detect the plasma circRNAs expression level, the Transcriptor First Strand cDNA Synthesis Kit (Roche, Basel, Switzerland) was used to generate cDNA from 1.5 μg of total RNA with an anchored-oligo (dT)15 primer and random primers based on the manufacturer’s instructions. GAPDH was used for circRNA template normalization. PCR was performed to detect the circRNA relative expression on an ABI 7,900 real-time PCR instrument (Applied Biosystems, Foster, CA, United States). The 2^–Δ^ ^Δ^ *^Ct^* methods were used to calculate the relative expression levels of circRNAs. PCR primers were shown as follows: hsa_circ_0070391 (F, CTCCATTCGGACTACCCCAAG; R, TAGCTTTGAGCCAACCTTCC), hsa_circ_0003935 (F, CCTTTGATGGGAGTTTGCCA; R, CAATCTGTTGTTGCCGCCTC), hsa_circ_0059880 (F, ATGCCGTCTACAAGGTTGGG; R, GAGCTGTCGATGAGGACCAC).

### Ablation Strategy and Left Atrial Voltage Matrix Mapping

All the AF indicated for radiofrequency catheter ablation were subjected to circumferential pulmonary vein isolation (CPVI) under local anesthesia and conscious sedation with fentanyl. The criterion for superior vena cava (SVC) isolation was based on the length of a SVC sleeve longer than 30 mm (13). The left atrial anterior wall was ablated with a low voltage area (LVA) in the anterior. AF with atypical left atrial flutter select mitral isthmus ablation. All the persistent AF selects the LA roof and LA posterior wall ablation except for conversion to sinus rhythm before operation and without low voltage in the left atrium. Paroxysmal AF was managed according to the ablation strategy of persistent AF if AF was still onset after pulmonary vein isolation.

The extent of left atrial fibrosis was calculated with the left atrial LVA by using the left atrial voltage matrix mapping in persistent AF. If the patient was still atrial fibrillation after CPVI, cardioversion was used to convert to sinus rhythm. If the first cardioversion failed to convert to sinus rhythm, then continue to cardioversion, if three times of cardioversion failed to convert to sinus rhythm, we will abandon the patient. The left atrial voltage matrix mapping was performed under sinus rhythm with a PENTARAY mapping catheter (PENTARAY catheter, Biosense Webster, Diamond Bar, CA). The three-dimensional mapping system (Carto-3, Biosense Webster) was used to map voltage points. The range of mapping voltage amplitude was set at 0.1–0.4 mV. The local electrogram, which was < 0.1 mV, was considered a LVA, representing the extent of left atrial fibrosis (5). The extent of left atrial LVA was calculated as a percentage of the LVA to the left atrial surface area.

### Statistical Analysis

Normally distributed data were presented as mean ± SD. Paired *t*-test, unpaired Student’s *t*-test, or non-parametric Mann–Whitney *U*-test was used to compare continuous variables between two groups. Two-way ANOVA with the Tukey test was performed to compare multiple groups. Categorical variables were presented as absolute or relative frequencies and compared using the chi-square test. Pearson’s correlation coefficients were used to analyze the correlation between the circRNA level and left atrial fibrosis. The value was log-transformed to normalize their distribution before statistical analysis. Moreover, ROC curve analysis was performed and the AUC was calculated to evaluate the candidate circRNAs’ diagnostic power. The heatmap package in R was used to construct the heatmap plot. Event-free survival was analyzed using Kaplan–Meier curves and compared by the log-rank test. The COX regression model was also used for survival analysis. The *p*-value of < 0.05 was considered to be statistically significant. The SPSS 17.0 (IBM, Armonk, NY, United States) and GraphPad Prism7.0 (San Diego, CA, United States) software were used for all statistical analyses and graph presentation, respectively.

## Results

### Identification of Three Different Expression Circular Ribonucleic Acids in Atrial Fibrillation

**FIGURE 1:**
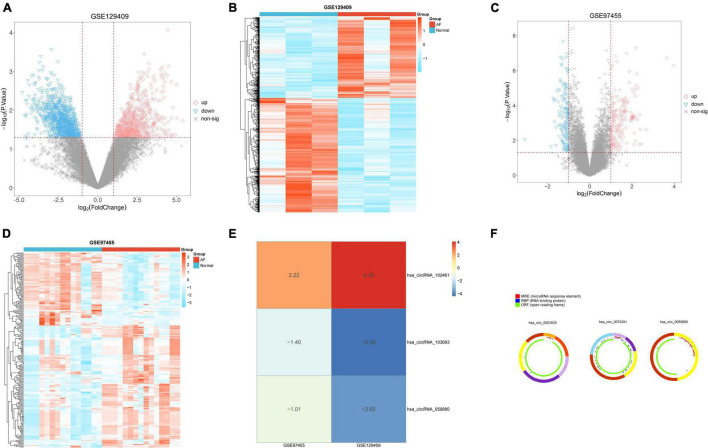
Identification of differentially expressed circRNAs (DECs) in two circRNA microarray datasets. **(A)** Volcano plot of 663 upregulated DECs and 940 downregulated DECs in the GSE129409 dataset; **(B)** the heatmap of DECs obtained from the GSE129409 dataset; **(C)** volcano plot of 142 upregulated DECs and 85 downregulated DECs in the GSE97455 dataset; **(D)** the heatmap of DECs obtained from the GSE97455 dataset; **(E)** the DECs of the two datasets were integrated and ranked with a robust method, the screened criteria were the *p* < 0.05, Three DECs were screened by integrating two datasets; and **(F)** the basic characteristics and structure patterns of these three circRNAs.

We analyzed the GSE129409 and GSE97455 circRNA microarray datasets via the limma package according to the log-folding variation values (| log_2_ FC| > 1 and *p* < 0.05). Based on the DEC results, a total of 663 significantly upregulated and 940 significantly downregulated DECs were confirmed in the GSE129409 dataset (Supplementary Table 6), and 227 DECs were filtered from the GSE97455 dataset (85 downregulated and 142 upregulated; Supplementary Table 6). DECs from the two circRNA microarray datasets were exhibited in volcano maps (Figures 1A,C) and heatmaps (Figures 1B,D). Furthermore, these DECs were integrated by the RRA method with a *p* < 0.05, and three robust DECs in GEO datasets were detected, of which 1 was upregulated (hsa_circRNA_102461) and two downregulated (hsa_circRNA_103693 and hsa_circRNA_059880) (Figure 1E). The basic characteristics and structural patterns of these three circRNAs are displayed in Table 1 and Figure 1F.

**TABLE 1:** The basic characteristics and structure patterns of these three circRNAs.

Agilent ID	circRNA ID	Position	Strand	Genomic	Gene	Regulation
				length	symbol	
Hsa_circRNA_102461	Hsa_circ_0070391	chr4:88099628–88106951	–	7,323	KLHL8	Up
Hsa_circRNA_103693	Hsa_circ_0003935	chr19:13443682–13445307	–	1,625	CACNA1A	Down
Hsa_circRNA_059880	Hsa_circ_0059880	chr20:32354656–32358098	+	3,442	ZNF341	Down

### Prediction of Circular Ribonucleic Acids–miRNA Interactions

**FIGURE 2:**
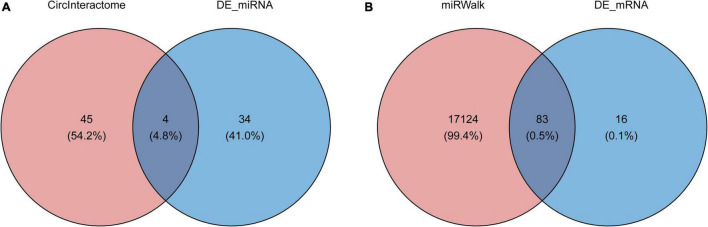
Prediction of circRNA-miRNA and miRNA-mRNA interactions. **(A)** The Venn diagram of 49 target miRNAs of circRNAs and 38 differentially expressed miRNAs screened from the GSE28954 dataset. **(B)** The Venn diagram of common target genes of the miRNAs was predicted by the miRWalk database and GSE115574 dataset.

To determine the function of the identified three robust DECs (hsa_circ_0003935, hsa_circ_0070391, and hsa_circ_0059880), their target miRNAs were predicted by informatics analysis based on the CircInteractome database. The results revealed that a total of 49 targeted miRNAs were predicted by the three DECs. Hsa_circ_0003935 predicted 7 targeted miRNAs (Supplementary Table 2); hsa_circ_0059880 predicted 13 targeted miRNAs (Supplementary Table 3); and hsa_circ_0070391 predicted 33 targeted miRNAs (Supplementary Table 4). Among them, hsa-miR-767-3p could be simultaneously targeted by both hsa_circ_0003935 and hsa_circ_0059880; hsa-miR-182 was the common target miRNA of hsa_circ_0003935 and hsa_circ_0070391; hsa_circ_0059880 and hsa_circ_0070391 could co-target hsa-miR-502-5p and hsa-miR-885-3p. Furthermore, a total of 38 differentially expressed miRNAs (DEMs; AF vs. control; | log_2_ FC| > 0.5 and *p* < 0.01; Supplementary Table 8) between AF (*n* = 10) and control (*n* = 18) groups were identified from the GSE28954 dataset by R package limma. To further obtain the AF-related targeting miRNAs, we performed an overlap analysis of the 49 predicted targeting miRNAs and 38 DEMs mentioned above (Figure 2A). Ultimately, four circRNA–miRNA pairs, including two circRNAs (hsa_circ_0003935 and hsa_circ_0070391) and four miRNAs (hsa-miR-223, hsa-miR-630, hsa-miR-21, and hsa-miR-1305) were identified (Supplementary Table 9). Additionally, we visualized the expression profiles of the identified four target DEMs in the GSE28954 dataset for AF and control groups and found that all three miRNAs were significantly overexpressed in AF except hsa-miR-1305, which was remarkably downregulated in the AF group (Supplementary Figure 1).

### Construction of the Circular Ribonucleic Acids–miRNA–mRNA Network

**FIGURE 3:**
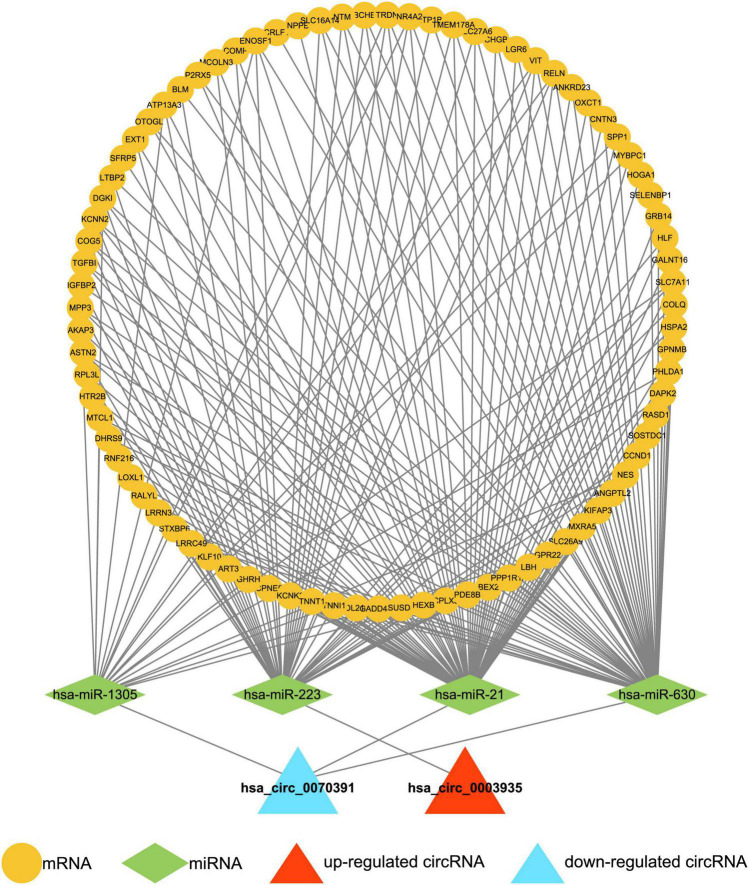
Construction of the circRNA-miRNA-mRNA network. The circRNA-miRNA-mRNA network was developed by combining circRNA-miRNA pairs and miRNA-mRNA pairs, including 2 circRNAs, 4 miRNAs, and 83 mRNAs.

A total of 17,207 target mRNAs were predicted for the four miRNAs in the above circRNA-miRNA axis through the miRWalk database (Supplementary Table 5). Meanwhile, 99 DEGs were identified from the GSE115574 dataset by R package limma (AF vs. control; | log_2_ FC| > 0.5 and *p* < 0.01; Supplementary Table 10). The 83 differentially expressed target mRNAs obtained from the intersection analysis (Figure 2B) formed 196 miRNA-mRNA relationship pairs (Supplementary Table 11) with the previous 4 target miRNAs. Besides, the expression patterns of the 83 differentially expressed target mRNAs in the GSE115574 dataset could be available in Supplementary Figure 2. Subsequently, a circRNA-miRNA-mRNA network was developed combining circRNA-miRNA and miRNA-mRNA pairs, which provided an overall perspective of regulation networks among 2 circRNAs (hsa_circ_0003935 and hsa_circ_0070391), 4 miRNAs (hsa-miR-223, hsa-miR-630, hsa-miR-21, and hsa-miR-1305) and 83 mRNAs (Figure 3). Specifically, hsa_circ_0003935 could competitively regulate the expression of 47 mRNAs with hsa-miR-223. Hsa_circ_0070391 could act as a sponge for hsa-miR-630, hsa-miR-21, and hsa-miR-1,305 to affect the expression of 57, 72, and 19 mRNAs, respectively. Among them, the expression of 11 mRNAs (TRDN, VIT, RELN, NR4A2, ENOSF1, DGKI, MTCL1, PDE8B, SLC7A11, MPP3, and SPP1) could be simultaneously affected by the competitive binding mode of hsa_circ_0070391 with hsa-miR-630, hsa-miR-21, and hsa-miR-1305.

### Gene Ontology and Kyoto Encyclopedia of Genes and Genomes Analysis of 83 Genes

**FIGURE 4:**
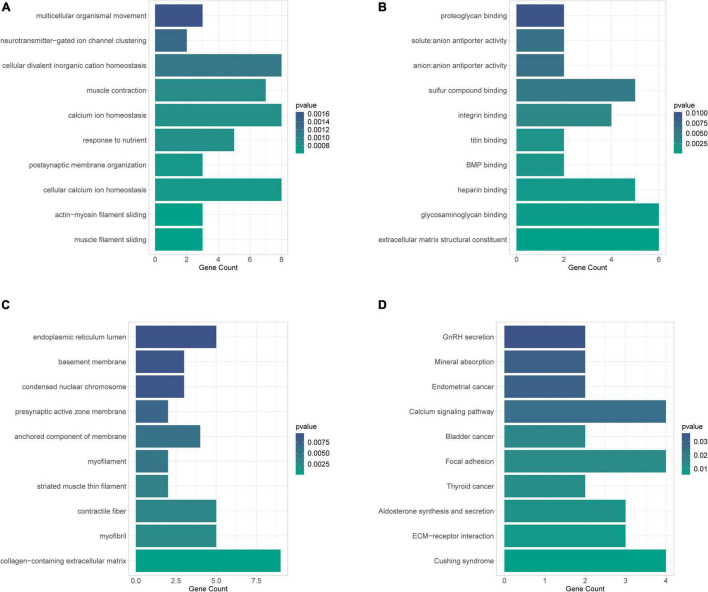
GO annotation and KEGG pathway analyses to obtain a primary understanding of the biological functions and pathways of target mRNA. **(A)** Bubble plot of BP; **(B)** bubble plot of MF; **(C)** bubble plot of CC; **(D)** top 10 enrichment of KEGG pathway analysis of DEGs.

To achieve a primary understanding of the biological functions and pathways of these genes, GO annotation and KEGG pathway analyses were conducted. GO analysis enriched a total of 183 terms in the biological process (BP) category (Supplementary Table 12), 30 terms in the cellular component (CC) category (Supplementary Table 13), and 38 terms in molecular function (MF) category (Supplementary Table 14), respectively. The top 10 GO-BP entries were illustrated in Figure 4A, where “muscle filament sliding.” “actin-myosin filament sliding,” and “cellular calcium ion homeostasis” were the three most enriched terms. Meanwhile, these mRNAs may perform MF such as “extracellular matrix structural constituent,” “glycosaminoglycan binding,” “heparin binding” (Figure 4B), CC such as “collagen-containing extracellular matrix” “myofibril” and “contractile fiber” (Figure 4C). A total of 20 pathways were enriched by KEGG analysis (Figure 4D and Supplementary Table 15). Among which the AF-related pathways such as ECM-receptor interaction, aldosterone synthesis and secretion, calcium signaling pathway, cortisol synthesis and secretion, Wnt signaling pathway, and p53 signaling pathway were significantly enriched.

### Prediction Results and Two-Dimensional Structure of Small Molecule Drugs

A total of 10 small molecule drugs were selected as potential drugs for AF treatment, of which, TTNPB, nystatin, Prestwick, and quinpirole could induce the expression of 83 target genes, while paroxetine, bufexamac, flunarizine, mercaptopurine, and spiperone could inhibit the expression of the genes and their two-dimensional structures are shown in Supplementary Figure 3.

### Corroboration of the Expression Level of Three Circular Ribonucleic Acids and miRNAs in the Plasma by Quantitative Reverse Transcription-Polymerase Chain Reaction

**FIGURE 5:**
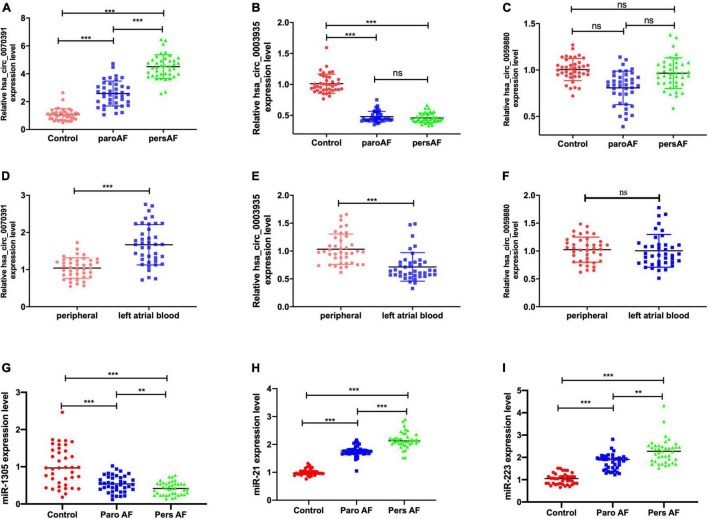
Corroboration of the expression level of the 3 circRNAs in plasma in AF by qRT-PCR. **(A)** The peripheral plasma hsa_circ_0070391 expression level in different types of AF and controls. **(B)** The peripheral plasma hsa_circ_0003935 expression level in different types of AF and controls. **(C)** The peripheral plasma hsa_circ_0059880 expression level in different types of AF and control. **(D)** The plasma hsa_circ_0070391 level in peripheral blood and left atrial blood. **(E)** The plasma hsa_circ_0003935 level in peripheral blood and left atrial blood. **(F)** The plasma hsa_circ_0059880 level in peripheral blood and left atrial blood. **(G)** The peripheral plasma has-miR-1305 expression level in different types of AF and controls. **(H)** The peripheral plasma has-miR-21 expression level in different types of AF and controls. **(I)** The peripheral plasma has-miR-223 expression level in different types of AF and controls. ***p < 0.001, **p < 0.01, ns: no significance. Paro AF, paroxysmal AF; Pers AF, persistent AF.

The baseline demographic characteristics and catheter ablation information of patients with 40 paroxysmal AF, 40 persistent AF, and 40 controls are shown in Table 2. We found the hsa_circ_0070391 expression level was increased in AF, whereas the hsa_circ_0003935 expression level was decreased, which was consistent with the screening results. However, the hsa_circ_0059880 expression level was similar between patients with AF and controls. Furthermore, the hsa_circ_0070391 expression level in persistent AF was higher than in paroxysmal AF (Figures 5A–C). Finally, we detected the expression level of the three circRNAs in left atrial blood and peripheral blood in AF, We found the expression level of hsa_circ_0070391 was higher in left atrial blood compared with peripheral blood in persistent AF, then the hsa_circ_0003935 expression was lower in left atrial blood compared with peripheral blood in persistent AF, whereas the hsa_circ_0059880 expression level was consistent (Figures 5D–F).

**TABLE 2:** Baseline characteristics of the AF and controls.

	Control (*n* = 40)	Paro AF (*n* = 40)	Pers AF (*n* = 40)	*P*-value
Age (year)	52 (47–65)	57 (49–70)	61 (52–69)	0.07
Male	18/40	22/40	22/40	0.59
LVEF (%)	66 ± 6	67 ± 4	61 ± 9	<0.001
LAD (mm)	34 ± 6	36 ± 5	41 ± 4	<0.001
LVDD (mm)	46 ± 5	47 ± 4	48 ± 5	0.06
BMI (kg/m^2^)	23.0 ± 2.6	23.0 ± 1.9	22.9 ± 1.9	0.07
Type 2 DM	7/40	7/40	4/40	0.56
Hyperlipidemia	10/40	6/40	10/40	0.46
Previous stroke	2/40	2/40	10/40	0.01
Hypertension	13/40	18/40	23/40	0.07
CHA2DS2-Vasc		1 (0–3)	2 (1–3)	0.25
0		12 (30%)	7 (17.5%)	0.40
1		9 (22.5%)	7 (17.5%)	
2		4 (10%)	10 (25%)	
3		8 (20%)	8 (20%)	
≥ 4		7 (17.5%)	8 (20%)	
Duration of atrial fibrillation (month)		12 (4–36)	6 (2–36)	0.26
**Medication history (%)**				
β-blockers	12	14	16	0.7
Antiarrhythmic drugs	5	9	14	
Digoxin	3	7	10	
**Fibrosis stage (left atrial LVA)**				
0–10%			28 (70%)	
10–20%			7 (17.5%)	
> 20%			5 (12.5)	
**Ablation strategy**				
PVI RF	/	40/40	40/40	
LA roof	/	2/40	38/40	
Mitral line	/	0	2/40	
CTI		3/40	33/40	
LA anterior wall		2/40	7/40	
LA posterior wall	/	2/40	38/40	
SVC isolation	/	29/40	15/40	

*Values are given as mean ± SD or n (%). LAD, left atrial diameter; LVEF, left ventricular ejection fraction; LVEDD, left ventricle end-diastolic diameter. LVA, low voltage area; LA, left atrial, PVI, pulmonary vein isolation; CTI, cavo-tricuspid isthmus; SVC, superior vena cava.*

Then the potential downstream miRNAs expression level of circRNAs was also detected in the plasma. The results showed that the has-miR-1305 expression level was decreased in the AF compared with controls in plasma, then has-miR-21 and has-miR-223 expression levels were increased (Figures 5G–I). However, the has-miR-630 expression level was consistent in AF patients and controls.

### Hsa_circ_0070391 Was Positively Associated With Left Atrial Fibrosis in Persistent Atrial Fibrillation

**FIGURE 6:**
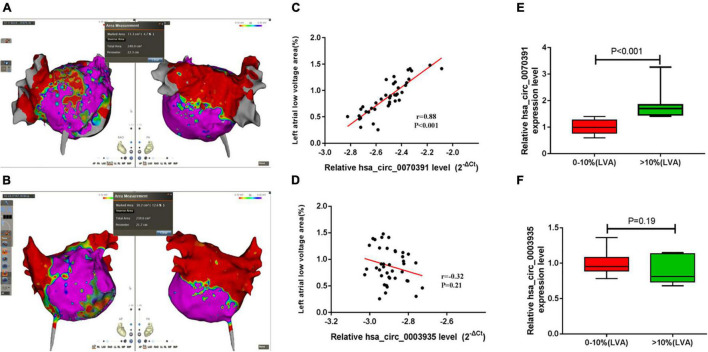
The correlation between different circRNAs and left atrial fibrosis in persistent AF. **(A,B)** The representative image of the left atrial low voltage area (LVA), red color represented the LVA (local electrograms < 0.4 mV), which was used to estimate the severity of left atrial fibrosis, Purple color represented the normal substrate (local electrograms > 0.4 mV). **(A)** Showed the low voltage area was less than 10%, **(B)** showed the low voltage area was more than 10%. Anterior–posterior angle position (left) and posterior–anterior position were simultaneous displayed (right); **(C)** the association between plasma hsa_circ_0070391 and extent of left atrial low voltage area; **(D)** the association between plasma hsa_circ_0003935 and extent of left atrial low voltage area; **(E)** the hsa_circ_0070391 expression level in different extents of left atrial fibrosis; **(F)** the hsa_circ_0003935 expression level in different extents of left atrial fibrosis.

The representative left atrial voltage matrix mapping is shown in Figures 6A,B. We also found the plasma hsa_circ_0070391 level was positively associated with left atrial fibrosis based on Pearson’s correlation coefficients (Figure 6C). However, hsa_circ_0003935 was not associated with left atrial fibrosis (Figure 6D). Furthermore, the extent of left atrial fibrosis was divided into two groups according to the LVA (LVA degree of < 10% and LVA degree of > 10%); the results showed the hsa_circ_0070391 expression level was higher in the LVA degree > 10% group than that of LVA degree < 10% (Figure 6E). However, the hsa_circ_0003935 expression level did not differ significantly between the two groups (Figure 6F).

### Hsa_circ_0070391 Was Associated With the Prognosis in Atrial Fibrillation With Radiofrequency Catheter Ablation

**FIGURE 7:**
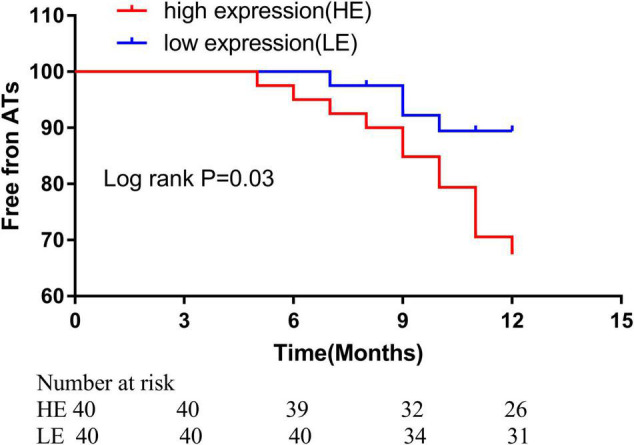
Kaplan–Meier freedom rate from ATa during different points after catheter ablation (high expression level: 70.0% vs. low expression level 90.0%, ratio: 3.05, 95%CI, 1.13–8.25, *p* = 0.04. ATa: atrial tachyarrhythmia.

We divided the AF patients into two groups according to the plasma hsa_circ_0070391 expression level, all the patients received the 24 h electrocardiogram. After catheter ablation, 36/40 (90.0%) of patients in the low expression group and 28/40 (70.0%) of patients in the high expression group remained free of atrial tachyarrhythmia at a mean 11.3 ± 1.7 months follow-up (*p* = 0.04). The log-rank test was shown to be in long-term atrial tachyarrhythmia-free survival (Figure 7). The COX regression analysis indicated that the difference expression of circRNA was the only independent predictor factor for AF prognosis (OR: 4.21; 95% CI, 1.054–10.147; *p* = 0.04).

### Diagnostic Value of Different Circular Ribonucleic Acids Expressions for Atrial Fibrillation

**FIGURE 8:**
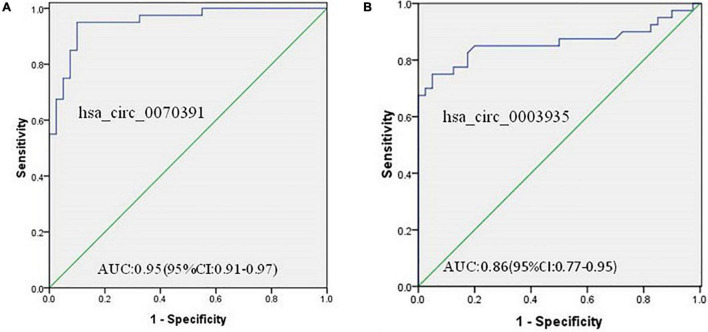
The diagnostic value of circRNAs for AF and diagnostic value to differentiate persistent from paroxysmal AF using the receiver operating characteristic (ROC) curve analysis. **(A)** The diagnostic value of hsa_circ_0070391 for AF and **(B)** the diagnostic value of hsa_circ_0003935 for AF.

ROC analysis was performed to demonstrate whether abnormal expressions of the circRNA (hsa_circ_0070391 and hsa_circ_0003935) have diagnostic values for AF. The results showed high expression of hsa_circ_0070391 and low expression of hsa_circ_0003935 could be differentiated in AF from controls with an AUC of 0.95 (95% CI, 0.91–0.97; 95% sensitivity and 90% specificity), and 0.86 (95% CI, 0.77–0.95; 70% sensitivity and 75% specificity), respectively (Figures 8A,B).

## Discussion

Atrial structural remodeling, characterized as atrial fibrosis, is the primary pathological mechanism for AF development and is associated with the prognosis. Therefore, seeking specific fibrosis biomarkers for AF is an urgent concern. This study demonstrated a circRNA–miRNA–mRNA network based on the publicly available gene microarray data. Two different circRNAs expressions were observed in the plasma between AF and controls. Furthermore, the hsa_circ_0070391 expression level in plasma was positively correlated with left atrial fibrosis in persistent AF, and the hsa_circ_0070391 also could serve as the prognosis biomarker for AF. It is noteworthy that since there are few available circRNA expression datasets from the peripheral blood datasets, we selected the circRNA expression datasets from plasma and heart tissues, respectively, to obtain DECs and construct ceRNA networks. However, it has been revealed that blood samples can be regarded as a surrogate tissue for the investigation of immune-related pathways in cardiovascular disease patients (14). Moreover, Steenman suggests that there is a variety of myocardial and vascular disease processes related to circulating cell types in blood, and the expression profile of plasma has been applied in plenty of cardiovascular diseases (15). Therefore, this study approach is an appropriate experimental method with scientific merit. However, further validation is necessary.

The ceRNA regulatory network has been proved to play an important role in the development of different cardiovascular diseases (16), such as AF (17). In this study, DEC, DEM, and DEG, which were closely related to AF, were identified using the circRNA microarray datasets from GSE129409 and GSE97455. Furthermore, by integrating the interaction between DEM and DEG or DEC, an AF-specific ceRNA-mediated network, including circRNA, miRNA, and mRNA was constructed. To further explore the potential biological function of circRNA in the occurrence of AF. The GO and KEGG analyses showed the target gene of the circRNA network was mainly involved in cellular calcium ion homeostasis and muscle contraction biological process, and was significantly associated with the calcium signaling pathway, focal adhesion, ECM-receptor interaction processes, and aldosterone synthesis and secretion. The previous studies have demonstrated that the cellular calcium handling dysfunction was involved in regulating atrial electrical remodeling, and inflammatory response, and multiple pathways can activate calcium channels (18). The excessive deposition of extracellular matrix (ECM) proteins in the myocardium were associated with cardiac fibrosis, which promoted the regression of arrhythmia and cardiac dysfunction (19). It is further suggested that the present circRNA–miRNA–mRNA ceRNA network may be involved in the occurrence and development of AF.

Previous studies have demonstrated that miR-21 expression was upregulated in AF and was associated with left atrial fibrosis (20). This study also found that miR-21 expression was increased in AF patients, which was consistent with previous studies. Moreover, the prediction potential downstream of miR-1305 of hsa_circ_0070391 was downregulated in AF patients. ceRNA mechanism showed that the circRNA expression was negatively correlated with downstream miRNA expression, further suggesting miR-1305 may be as downstream of hsa_circ_0070391.

circRNA can be stably expressed in the blood or tissues owing to their resistance to RNA exonucleases or RNase R and special closed-loop structure (21). Studies have shown that abnormal expression of circRNAs in the serum or plasma could be used as non-invasive diagnostic and prognostic biomarkers for cardiovascular diseases (22, 23). Furthermore, reports have demonstrated that the circRNA was differentially expressed in the peripheral blood and atrial tissues in AF and could regulate atrial remodeling (24–26). However, the role of circRNAs remains to be further explored in AF. In this study, we verified plasma hsa_circ_0070391 expression level was upregulated and hsa_circ_0003935 was downregulated in AF. Furthermore, the plasma level of hsa_circ_0070391 was significantly increased in persistent AF than in paroxysmal AF, suggesting that hsa_circ_0070391 plays a major role in the progression of AF and was associated with the AF duration. In addition, the high expression of hsa_circ_0070391 increased the recurrence rate after AF catheter ablation. These results further suggest that hsa_circ_0070391 dynamically changes during different AF stages. Therefore, hsa_circ_0070391 may serve as a potential prognosis biomarker for AF.

Hsa_circ_0070391 is a non-coding RNA transcribed by KLHL8. Previous studies have shown that circ-KLHL8 could be used as a candidate biomarker for the diagnosis of diabetes and promote epithelial healing, regulating endothelial cell apoptosis, survival, and maintaining endothelial function through activating the miR-212-3p/SIRT5 signaling pathway (27). There is no relevant report of hsa_circ_0070391 in the field of cardiovascular disease. The hsa_circ_0003935 expression was transcribed by CACNA1A, which has not been reported in cardiovascular diseases. The two differentially expressed circRNAs were first reported in this study.

The DECAAF I study demonstrated that the degree of atrial fibrosis determined by late gadolinium enhancement magnetic resonance imaging (LGE-MRI) is related to the recurrence of catheter ablation in patients with AF (28). Moreover, the severity of atrial fibrosis is also associated with the AF course (29). The latest DECAAF II study demonstrated that atrial fibrotic ablation under the guidance of cardiac enhanced MRI with pulmonary vein electrical isolation could improve the effectiveness of catheter ablation in the treatment of persistent AF with < 20% degree of fibrosis as compared with simple pulmonary vein electrical isolation (30). A previous study also reported that the reduction of atrial fibrosis through radiofrequency catheter ablation could improve the success rate of AF catheter ablation and enhance sinus rhythm maintenance (31). The administration of drugs such as angiotensin receptor blockers or angiotensin-converting enzyme inhibitors to prevent atrial fibrosis could reduce the incidence of AF and improve the prognosis (32). Therefore, seeking early atrial fibrosis biomarkers is an effective method to improve the prognosis of AF. Furthermore, it can identify high-risk recurrence in AF after radiofrequency catheter ablation and serve as a guide for early intervention. This study demonstrated that the plasma hsa_circ_0070391 expression level was positively associated with left atrial fibrosis and dynamic changes in different courses of AF. More importantly, left atrial substance abnormalities were more correlated with the occurrence of AF than right atrial. The present results showed the expression level of hsa_circ_0070391 was increased in left atrial blood than in peripheral blood. These results suggest that hsa_circ_0070391 could serve as a sensitive biomarker for predicting atrial fibrosis.

However, the expression of hsa_circ_0003935, which was transcribed by CACNA1A, was decreased in AF. The association between hsa_circ_0003935 and left atrial fibrosis was without significance. Previous studies have shown the CACNA1A is down-regulated in AF and involved in regulating atrial electrical remodeling (33). And atrial electrical remodeling occurred in the early stage of atrial remodeling before atrial fibrosis. So we speculate hsa_circ_0003935 may be related to atrial electrical remodeling instead of structural remodeling.

### Limitations

This study also has three limitations. First, a larger sample size is required to further verify the association between left atrial fibrosis and circRNAs. More methods are required to evaluate left atrial fibrosis such as cardiac magnetic resonance imaging (28). Finally, the circRNA function during AF development *in vivo* and *in vitro* was not investigated. Therefore, these limitations should also be investigated in the future.

## Conclusion

In conclusion, this study identified a circRNA–miRNA–mRNA network which was closely associated with AF. Furthermore, we also demonstrated that the plasma hsa_circ_0070391 was associated with left atrial fibrosis and prognosis with AF with radiofrequency catheter ablation, which may become a new molecular target for AF in the future.

## Data Availability Statement

The datasets presented in this study can be found in online repositories. The names of the repository/repositories and accession number(s) can be found in the article/Supplementary Material.

## Ethics Statement

The studies involving human participants were reviewed and approved by The First People’s Hospital of Yunnan Province. The patients/participants provided their written informed consent to participate in this study.

## Author Contributions

JF and FW: conceptualization and funding acquisition. FW, XZ, XK, XG, and JW: methodology. FW: software. XZ and XG: formal analysis. JF: writing—original draft preparation and project administration. All authors have read and agreed to the published.

## Conflict of Interest

The authors declare that the research was conducted in the absence of any commercial or financial relationships that could be construed as a potential conflict of interest.

## Publisher’s Note

All claims expressed in this article are solely those of the authors and do not necessarily represent those of their affiliated organizations, or those of the publisher, the editors and the reviewers. Any product that may be evaluated in this article, or claim that may be made by its manufacturer, is not guaranteed or endorsed by the publisher.

## Footnotes

1
https://www.ncbi.nlm.nih.gov/

2
https://circinteractome.nia.nih.gov/

3
http://mirwalk.umm.uni-heidelberg.de/

4
https://cytoscape.org/

5
https://portals.broadinstitute.org/cmap

6
https://pubchem.ncbi.nlm.nih.gov/
