# Supplementary material for: Integrated Analysis of circRNA-miRNA-mRNA-Mediated Network and Its Potential Function in Atrial Fibrillation
Source: Front Cardiovasc Med. 2022 Jun 30;9:883205. doi: 10.3389/fcvm.2022.883205 (PMC9279703; doi:10.3389/fcvm.2022.883205)
Supplement: Supplementary file 1 [file Data_Sheet_1.pdf]

Supplementary Material

Supplement Tables and Figures

Table S1: 4 microarray expression profile data sets from the public functional genomics database.

Table S2-4: The target miRNAs of the DECs were predicted by a web tool circular RNA Interactome.

Table S5: The miRWalk (http://mirwalk.umm.uni-heidelberg.de/) database was used to predict the mRNAs of the overlapped miRNA.

Table S6: The different expression circRNA in the GSE129409 dataset.

Table S7: The different expression circRNA in the GSE97455 dataset.

Table S8: The target miRNAs of circRNAs and interacted with 38 DEMs in the GSE28954 dataset.

Table S9: Prediction of circRNA-miRNA interactions.

Table S10: Construction of circRNA-miRNA-mRNA network.

Table S11: The differentially expressed target mRNAs obtained from the intersection analysis formed 196 miRNA-mRNA relationship pairs with the previous 4 target miRNAs

Table S12: GO analysis enriched a total of 183 terms in the BP category

Table S13: GO analysis enriched a total of 30 terms in the CC category

Table S14: GO analysis enriched a total of 38 terms in the MF category

Table S15: KEGG analysis

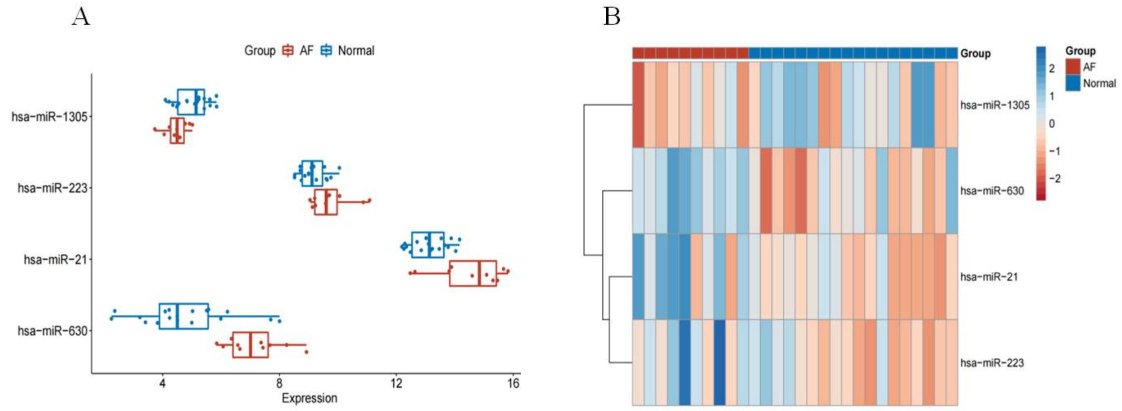

Figure S1: The visualized expression profiles of the target DEMs in the GSE28954 dataset for AF and control groups. **A**: The expression level of the differentially miRNAs; **B**: The expression level of the differentially miRNAs illustrated with heatmap.

A B

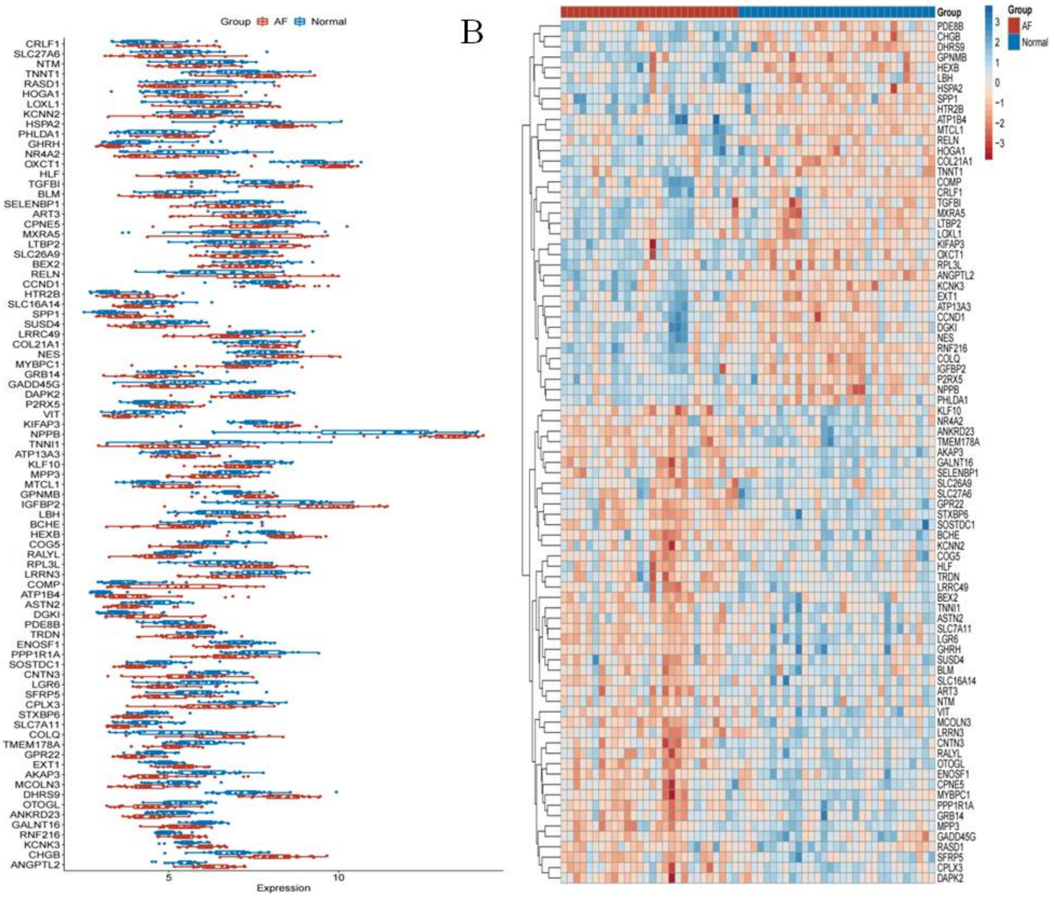

Figure S2: The expression patterns of the 83 differentially expressed target mRNAs in the GSE115574 dataset.

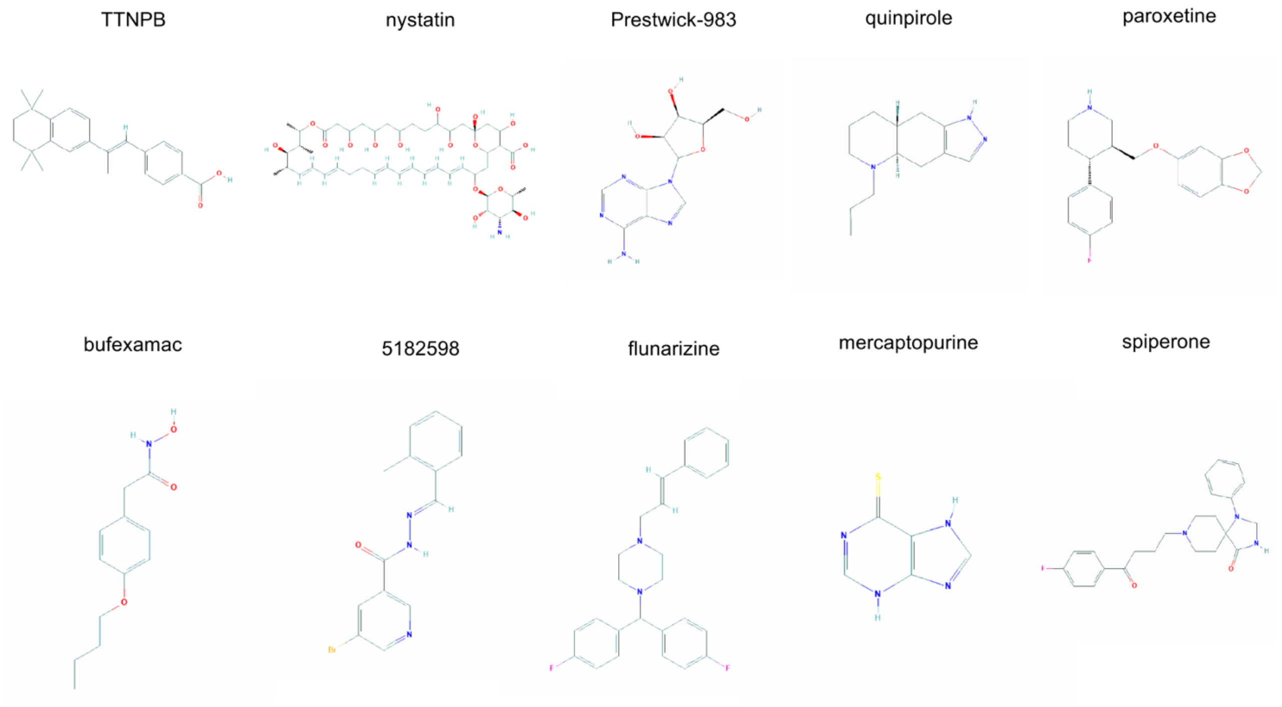

FigureS3: Small-molecule structure of the predicted drugs in CMap

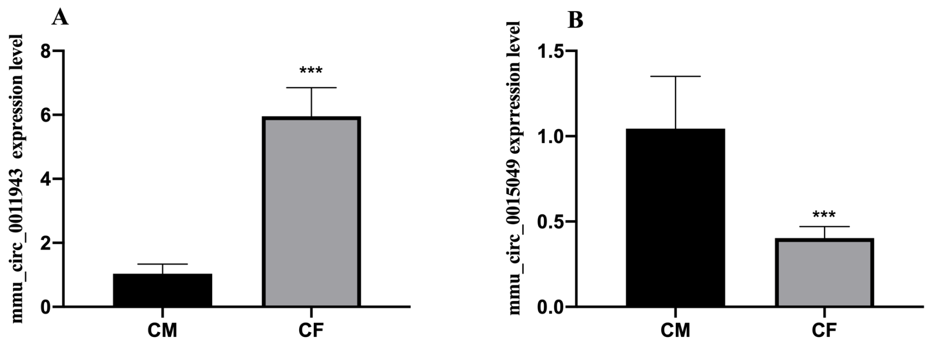

Figure S4: The difference expression of circRNAs in fibroblast and cardiomyocyte in mouse atrial tissue. **A**: The difference expression level of mmu_circ_0011943 was detected in fibroblast (CF) and cardiomyocyte (CM) (n=6, **P<0.001). **B**: The difference expression level of mmu_circ_0015049 was detected in fibroblast and cardiomyocyte. **P<0.001. GAPDA was used as internal control.
